# Neuroglobin mediates neuroprotection of hypoxic postconditioning against transient global cerebral ischemia in rats through preserving the activity of Na^+^/K^+^ ATPases

**DOI:** 10.1038/s41419-018-0656-0

**Published:** 2018-05-25

**Authors:** Haixia Wen, Liu Liu, Lixuan Zhan, Donghai Liang, Luxi Li, Dandan Liu, Weiwen Sun, En Xu

**Affiliations:** 1 Institute of Neurosciences and Department of Neurology of the Second Affiliated Hospital of Guangzhou Medical University and Key Laboratory of Neurogenetics and Channelopathies of Guangdong Province and the Ministry of Education of China, Guangzhou, 510260 P. R. China; 2 Department of Environmental Health, Rollins School of Public Health, Emory University, 1518 Clifton Road, 2030B, Atlanta, GA 30322 USA

## Abstract

Hypoxic postconditioning (HPC) is an innovative neuroprotective strategy with cytoprotective effects on the hippocampal neurons against transient global cerebral ischemia (tGCI) in adult rats. However, its molecular mechanisms have not yet been adequately elucidated. Neuroglobin (Ngb) is an endogenous neuroprotectant with hypoxia-inducible property, and its role in experimental stroke has been increasingly attractive. Hence, the purpose of this study is to explore the involvement of Ngb in HPC-mediated neuroprotection and to further investigate its underlying molecular mechanism. We found that HPC increased Ngb expression in CA1 subregion after tGCI. Also, the inhibition of Ngb expression with Ngb antisense oligodeoxynucleotide (AS-ODNs) eliminated the neuroprotective effect mediated by HPC, whereas overexpression of Ngb ameliorated neuronal damage in CA1 after tGCI, indicating that HPC conferred neuroprotective effects via upregulation of Ngb. We further showed that HPC increased the membranous level of Na^+^/K^+^ ATPases β1 subunit (Atp1b1) in CA1 after tGCI. Furthermore, we demonstrated that Ngb upregulation in CA1 after HPC maintained the membranous level of Atp1b1 through Ngb–Atp1b1 interaction and reduced the glutathionylation of membranous Atp1b1 via suppression of reactive oxygen species (ROS), ultimately preserving the activity of NKA. Taken together, these data indicate that Ngb is involved in the neuroprotection of HPC against tGCI via maintenance of NKA activity in the hippocampal CA1.

## Introduction

Transient global cerebral ischemia (tGCI) may result from cardiac arrest, serious hypertension, asphyxia, shock, or brain injuries, etc. The hippocampal CA1 pyramidal neurons are essential for cognitive functions and are selectively vulnerable to tGCI^1,2^. Although tremendous work has been directed to the molecular basis of the selective vulnerability of CA1, until now, there have been few effective therapies to this delayed neuronal damage after brain ischemia/reperfusion. Previously, we had demonstrated that hypoxic postconditioning (HPC) with 8% O_2_ for 60–120 min significantly reduced cell death in the CA1 subregion after 10 min of tGCI. HPC was effective only when applied 1–2 days after ischemia. The maximum protective effect of HPC was achieved with 120 min of hypoxia and 1-day interval between hypoxia and tGCI^3^. HPC has attracted great attention, since it holds the promise as an innovative endogenous neuroprotective strategy against tGCI^44,5^. Despite the identification of several triggers and mediators of HPC^3,6–8^, the precise mechanisms by which HPC protects the brain against tGCI remain poorly understood.

Neuroglobin (Ngb) was originally identified in the human and the mouse brain. It is predominantly expressed in the neurons and the astrocytes^9^. Sharing little similarity in the amino-acid sequence, Ngb is distinct from myoglobin and hemoglobin in evolution and function^10^. Ngb may serve as a hypoxic sensor and initiate signal transduction involving in oxidative and hypoxic pathway, cell survival/apoptotic pathway, and ATP pathway in neuronal cells^11^. It has also been proposed that the neuroprotection of Ngb at the mitochondrial level is not only related to its activities to scavenge a variety of free radicals, but also to its ability to interact with cytochrome C (Cyc) in the mitochondria^12,13^. In recent years, the neuroprotective role of Ngb, particularly in hypoxic and ischemic conditions, has been explored^14–16^. Li et al. demonstrated that limb ischemic preconditioning upregulates Ngb in the hippocampal CA1 to diminish mitochondrial dysfunction induced by tGCI^17^. Similar result was also reported in vitro that the overexpression of Ngb could suppress ROS production and ameliorate the neurotoxicity induced by oxygen-glucose deprivation (OGD) in primary cortical neurons^18^. However, the neuroprotection of Ngb upregulation in cerebral ischemia was challenged by a few studies^19,20^. Research from Raida et al. revealed that the infarct size was reduced in Ngb-null mice after permanent MCAO, compared to the wild-type mice^20^. In fact, both ischemia and hypoxia exhibit regulatory functions in Ngb induction, leading to either promotion or suppression of Ngb expression in different tissues and distinct cell types^21,22^. To fill in these knowledge gaps, we examined the expression of Ngb after tGCI with or without hypoxia in CA1. Afterwards, we tested whether the alternation of Ngb expression is associated with the neuroprotective effect mediated by HPC after tGCI.

Na^+^/K^+^ ATPases (NKA), constituted from a catalytic α (α1, α2, α3, and α4) subunit and a regulatory β (β1, β2, and β3) subunit, has been proven to be a vital energy transducing ion pumps in maintaining intracellular ion homeostasis and excitability^23,24^. The dysfunction of the ionic pumps, particularly the NKA, fails to maintain the ionic gradient across the neuronal membrane, resulting in massive cytoplasmic Na^+^ and Ca^2+^ accumulation, swelling, and degeneration of organelles, and eventually leading to cell dissolution after ischemia^25–27^. No less importantly, the β subunit is critical for the ion transport activity and the modulation of K^+^ and Na^+^ affinity of NKA^28,29^. Among the three isoforms of β subunit, Na^+^/K^+^ ATPases β1 subunit (Atp1b1) is one of the Ngb-interacting proteins recently identified by the yeast two-hybrid screening system that has the highest positive clone number^30^. Atp1b1 contains seven cysteine (Cys) residues. Six of them locate in the extracellular domain^31^, only Cys-46 locates in the lipid bulk phase of the membrane and is expected to prevent from accessing to the cytosolic, hydrophilic glutathione^32^. Additionally, oxidative stress can induce a reversible suppression on the NKA activity^33–35^, which is mediated by glutathionylation of its β1 subunit at Cys-46^36^. Intriguingly, as a scavenger of nitric oxide and reactive oxygen species (ROS), Ngb plays an important role against oxidative stress^13,37^. Therefore, Ngb is likely to be associated with the regulation of Atp1b1 directly or indirectly, which might explain how Ngb involves in the neuroprotection of HPC against tGCI.

Considering the hypoxia-inducible feature and the antioxidant property of Ngb, we hypothesized that the alteration of Ngb in CA1 subregion contributes to the neuroprotection of HPC against tGCI by reducing the ROS production, subsequently upregulating membranous Atp1b1, suppressing the glutathionylation of Atp1b1, and eventually preserving the activity of NKA.

## Results

### The upregulation of Ngb induced by HPC in the hippocampal CA1 contributed to the neuroprotection against tGCI

**Fig. 1:**
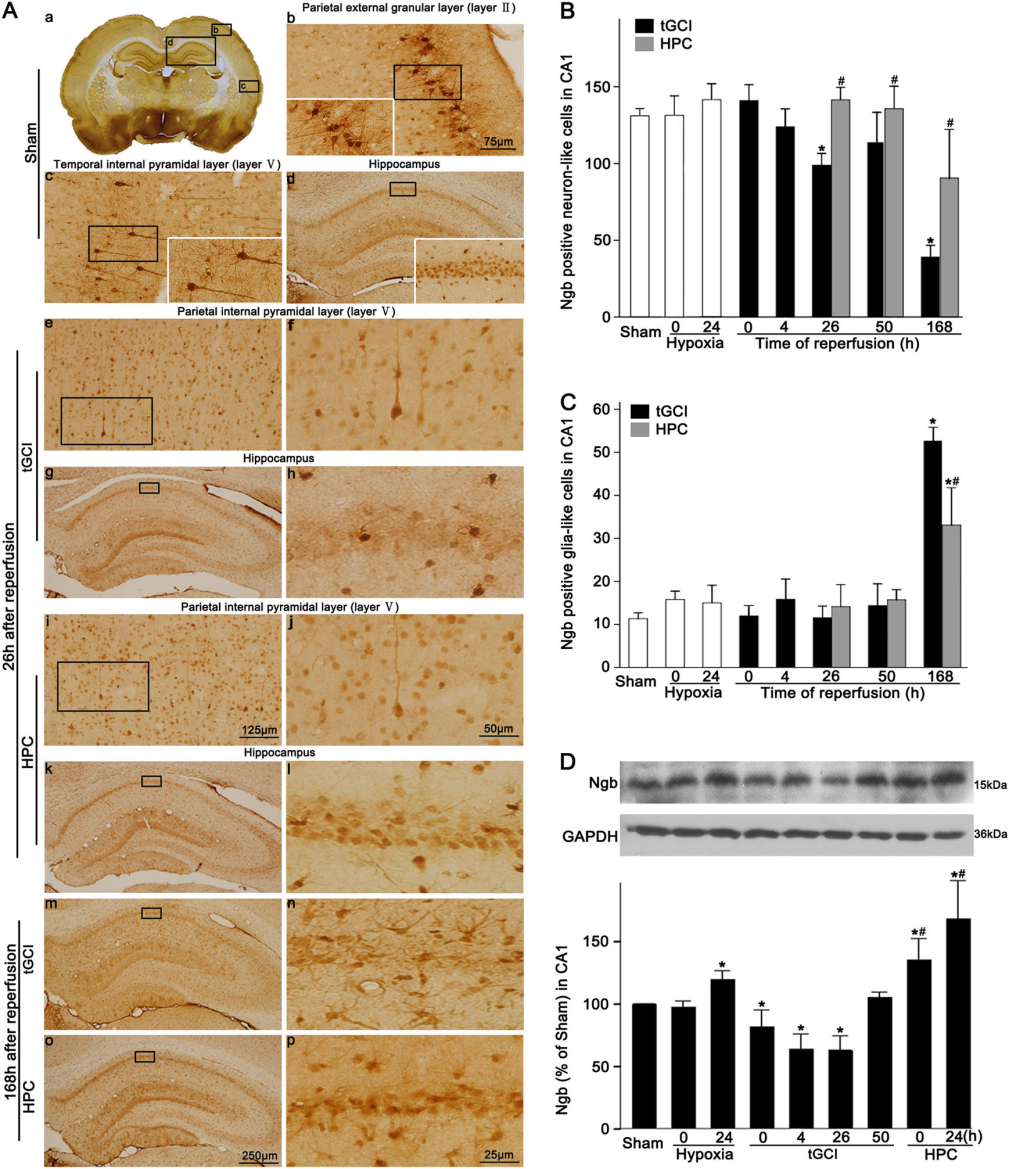
Effect of HPC on the protein expression of Ngb in CA1 after tGCI. **A** Immunohistochemistry of Ngb in the rat brains. Representative images show Sham-operated group (a–d), 26 h after reperfusion of the tGCI groups (e–h), 26 h after reperfusion of the HPC groups (i–l), 168 h after reperfusion of the tGCI groups (m, n), and 168 h after reperfusion of the HPC groups (o, p), respectively. Ngb-positive cells were observed in the external granular layer of parietal cortex (b), internal pyramidal layer of the temporal cortex (c) and parietal cortex (e, f, i, j), and pyramidal cell layer of the hippocampus (d, g, h, k–p). Scale bar: d, g, k, m, o: 250 μm; b, c: 75 μm; e, i: 125 μm; f, j: 50 μm; h, l, n, p: 25 μm. **B**, **C** Quantitative analysis of Ngb-positive neuron-like cells and glia-like cells in the CA1, respectively (*n* ≥ 6 in each group). **D** Western blot analysis of Ngb in CA1. The histogram presents the quantitative analyses of Ngb levels (*n* = 3 in each group). Data are expressed as percentages of value of Sham-operated animals. Each bar represents the mean ± S.D. *p < 0.05 vs. Sham-operated animals and ^#^*p* < 0.05 vs. tGCI group at the same time point

**Fig. 2:**
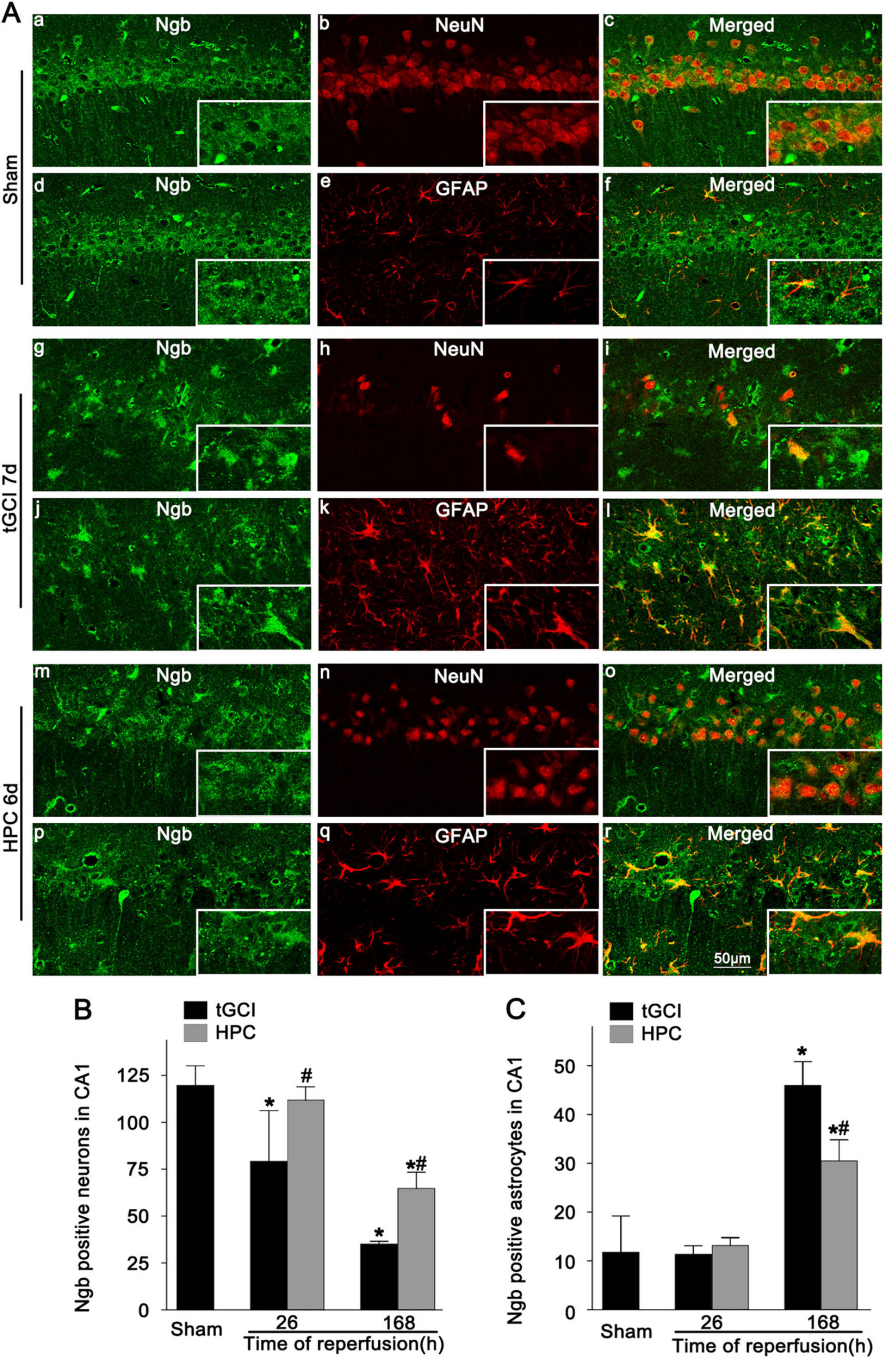
Cellular localization of Ngb in the CA1 after tGCI with or without HPC. **A** Representative photomicrographs of fluorescent double staining show that Ngb (green) is mainly distributed in the cytoplasm and the surrounded NeuN (red)-positive nuclei in CA1 (a–c) with a few colocalization of Ngb (green) with GFAP (red) (d–f) in the Sham-operated group. However, Ngb (green)-positive cells were mainly colocalized with GFAP (red) (j–l) rather than NeuN (red) (g–i) in the CA1 at 168 h after tGCI. Alternatively, Ngb (green) located mainly in NeuN (red) (m–o) and partially in GFAP-positive cells (p–r) in the CA1 at 168 h after HPC. Scale bar: 50 μm. **B**, **C** Quantitative analysis of Ngb-positive neurons and Ngb-positive astrocytes in the CA1, respectively (*n* ≥ 3 in each group). Each bar represents the mean ± S.D. *p < 0.05 vs. Sham-operated animals and ^#^*p* < 0.05 vs. tGCI group at the same time point

Immunohistochemical assay was conducted to examine the distribution of Ngb in the brain. The results showed a wide distribution of Ngb-positive cells in the Sham brains, including cerebral cortex, hippocampus, and subcortical structures (Fig. 1A, a–d). These cells have deep staining nuclei with several short dendrites in the parietal external granular layer (layer II) (Fig. 1A, b) and large soma with elongated axon in the temporal internal pyramidal layer (layer V) (Fig. 1A, c). Similar cells were also found in the parietal internal pyramidal layer (layer V) of tGCI and HPC rats at 26 h after reperfusion (Fig. 1A, e, f, i, j). In CA1, Ngb-positive cells mainly existed in the pyramidal cell layer with round and regular nuclei (Fig 1A, d). Double-fluorescent immunohistochemistry revealed that they were neuronal nuclei (NeuN)-positive (Fig. 2A, a–c) and only a few were colocalized with glial fibrillary acidic protein (GFAP) (Fig. 2A, d–f), indicating predominant neuronal localization of Ngb in the Sham brains. Notably, Ngb was expressed mainly in cells with polymorphic somata and processes. These cells were dispersedly distributed in the stratum radiatum and the oriens layer at 168 h of reperfusion after tGCI (Fig. 1A, m and n). The double-labeled immunofluorescence studies further revealed that these cells were mainly GFAP-positive (Fig. 2A, g–l). In contrast, the majority of Ngb-positive cells in HPC rats exhibited similar appearance to neurons (Fig. 1A, o and p). Also, these cells were shown to be NeuN-positive (Fig. 2A, m–o). The quantitative analysis showed that the number of Ngb-positive neuron-like cells in CA1 decreased at 26 h, then increased to the basal expression level at 50 h, and significantly reduced at 168 h after tGCI, while kept increasing consistently in the HPC groups (Fig. 1B). Meanwhile, Ngb-positive glia-like cells sharply increased at 168 h of reperfusion after tGCI, instead of the early phase of reperfusion, and a reverse trend was observed in the HPC group (Fig. 1C). These observations were further confirmed by the colocalization of Ngb with NeuN or GFAP (Fig. 2B, C). Also, Ngb level gradually decreased at 0–26 h after reperfusion of tGCI and increased back to the basal level at 50 h, while in the HPC groups, Ngb expression dramatically increased right after ischemia (Fig. 1D).

**Fig. 3:**
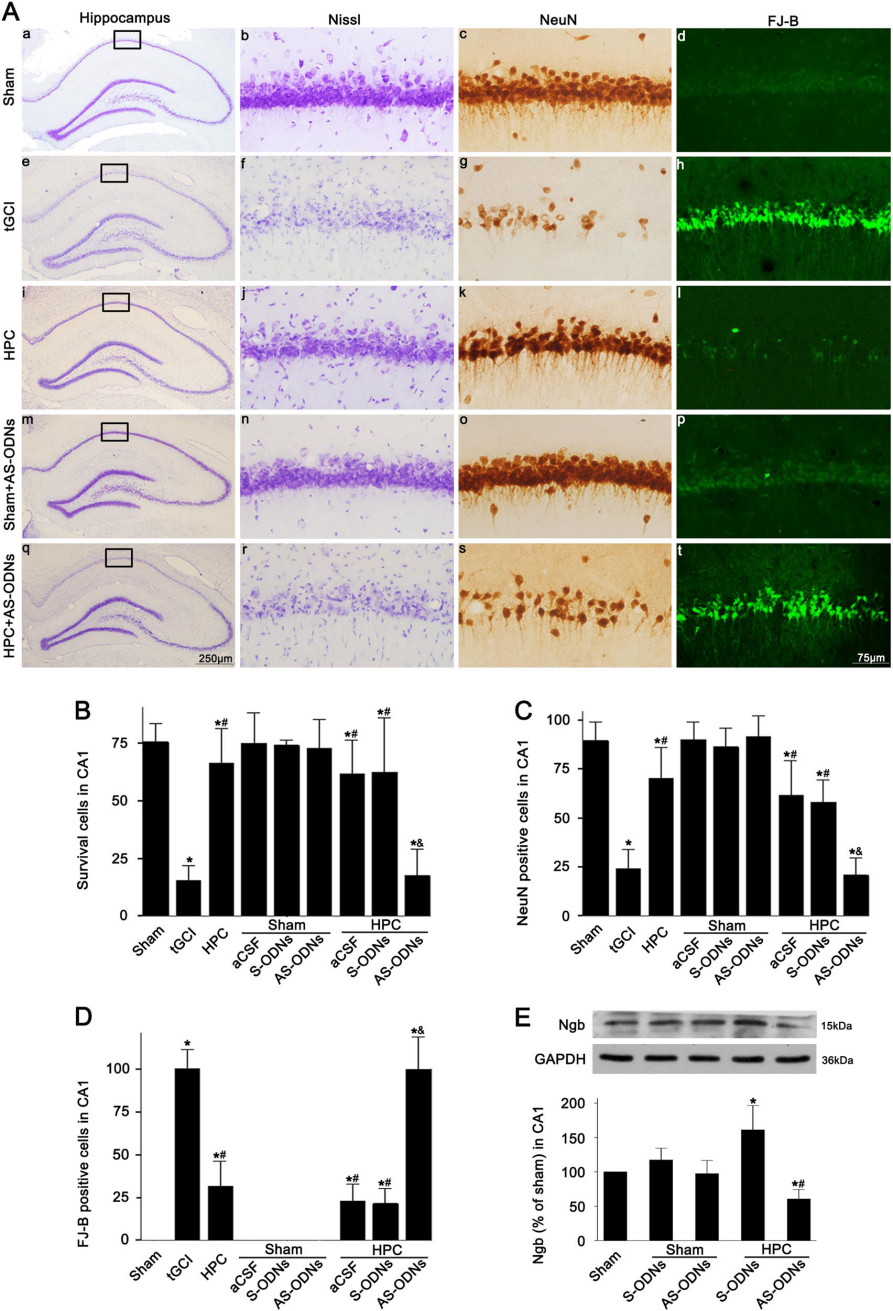
Effects of Ngb knockdown on the protein expression of Ngb and neuronal cells damage in CA1 after ischemia with or without HPC. **A** Representative microphotographs of cresyl violet staining, NeuN immunostaining, and FJ-B staining in the hippocampus at 7 days after tGCI with or without ODNs administration. Sham group (a–d); tGCI group (e–h); HPC group (i–l); Sham + AS-ODNs group (m–p), injection with AS-ODNs without ischemia or hypoxia; HPC + AS-ODNs group (q–t), injection with AS-ODNs before tGCI and HPC. Scale bar: a, e, i, m, q: 250 μm, b–d, f–h, j–l, n–p, r–t: 75 μm. **B**–**D** Quantitative analyses of survival cells, NeuN-positive cells and FJ-B-positive cells in the CA1 (*n* = 6 in each group). Each bar represents the mean ± S.D. *p < 0.05 vs. Sham-operated group, ^#^*p* < 0.05 vs. tGCI group, and ^&^*p* < 0.05 vs. HPC group. **E** Effects of Ngb ODNs on the expression of Ngb in the CA1 of Sham-operated and HPC rats using immunoblot analysis at 26 h after reperfusion (*n* > 3 in each group). The values are expressed as mean ± S.D. *p < 0.05 vs. Sham-operated animals and ^#^*p* < 0.05 vs. HPC group with S-ODNs administration

Next, Ngb antisense oligodeoxynucleotide (AS-ODNs) or Ngb-carried lentivirus was utilized to further confirm the neuroprotective role of Ngb mediated by HPC. As shown in Fig. 3, intraventricular administration of Ngb sense oligodeoxynucleotide (S-ODNs) or AS-ODNs had no impact on the Ngb expression (Fig. 3E) or on the pyramidal neurons in CA1 of the Sham-operated rats (Fig. 3A, m–p; Fig. 3B, C). However, the administration of Ngb AS-ODNs, rather than S-ODNs, significantly reduced the Ngb expression after HPC (Fig. 3E). Also, compared with HPC rats, the neuronal damage was markedly aggravated in the CA1 of HPC rats that were preadministrated with Ngb AS-ODNs (Fig. 3A, q–t), accompanied by a decrease in surviving and NeuN-positive cells, as well as an increase in Fluoro-Jade B (FJ-B)-positive cells (Fig. 3B–D).

**Fig. 4:**
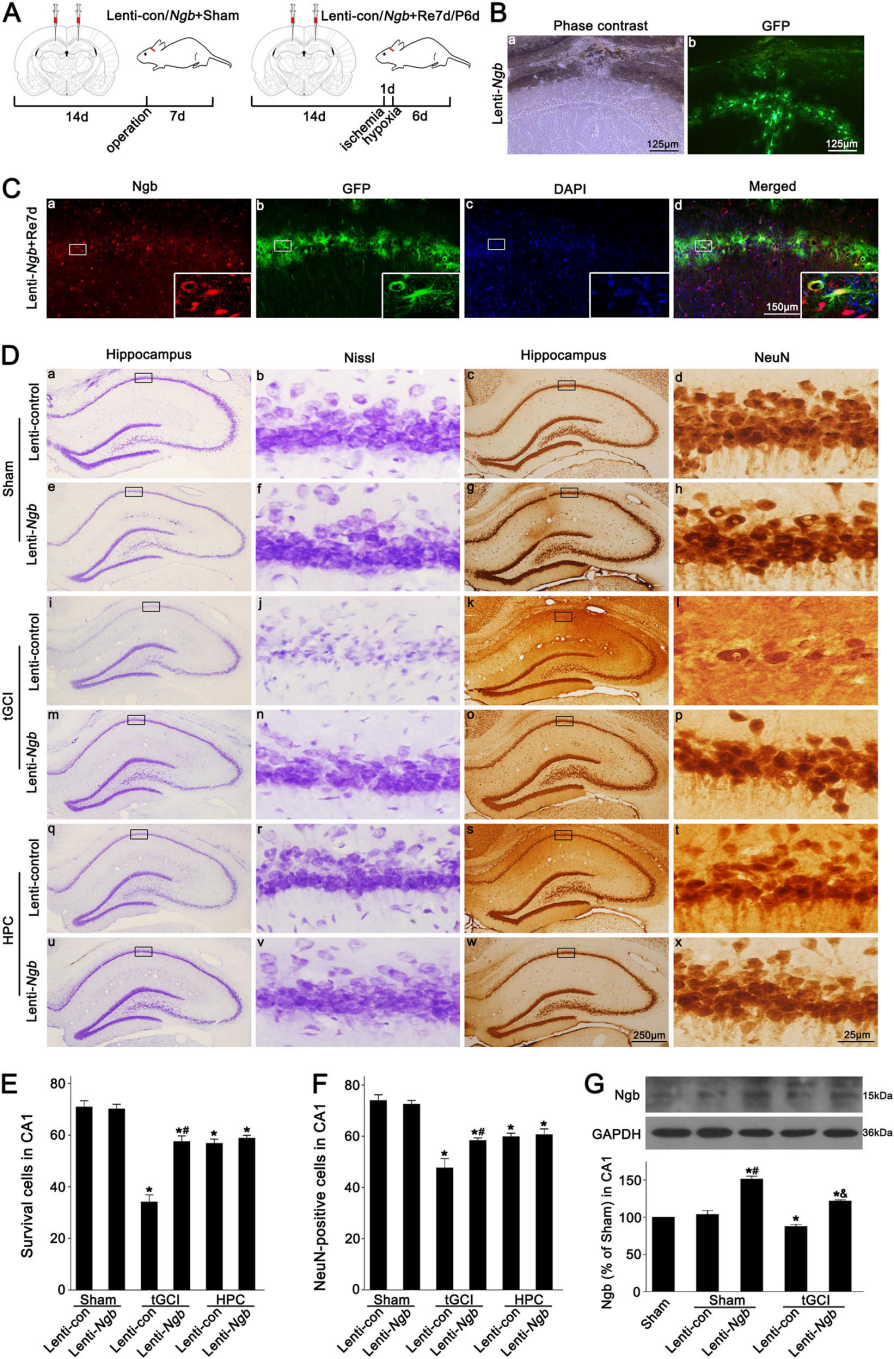
Effect of Ngb overexpression on Ngb expression and neuronal cells damage in CA1 after ischemia with or without HPC. **A** Design of experiments in which rats were stereotaxically injected bilaterally with Ngb lentiviral vectors in the dorsal CA1 pyramidal layer and subjected to either Sham-operation or tGCI with or without hypoxia. **B** Phase contrast and fluorescent images from the coronal sections of CA1 following injection of Ngb lentiviral vectors in Sham-operated animals. **C** Representative photomicrographs show the co-localization of Ngb (red), GFP (green), and 4′,6-diamidino-2-phenylindole (DAPI) (blue) in the brain sections from animals at 7 days after tGCI with Lenti-*Ngb* injection. **D** Cresyl violet stained and NeuN-immunostained hippocampal sections from rats administered bilaterally with either Lenti-control or Lenti-*Ngb* at 7 days after reperfusion with or without hypoxia. Boxes indicate the magnified regions displayed in the right panel. **E**, **F** Quantitative analyses of the survival cells and NeuN-positive cells in the CA1. Each bar represents the mean ± S.D. *p < 0.05 vs. Sham-operated animals with injection of Lenti-control, ^#^*p* < 0.05 vs. tGCI group with injection of Lenti-control (*n* = 6 in each group). **G** Representative immunoblots of Ngb expression in the CA1. The histogram presents the quantitative analyses of Ngb levels. Data are expressed as percentage of value of the Sham-operated animals. Each bar represents the mean ± S.D. *p < 0.05 vs. Sham-operated animals, ^#^*p* < 0.05 vs. Sham-operated group with injection of Lenti-control, and ^&^*p* < 0.05 vs. HPC group with injection of Lenti-control (*n* ≥ 3 in each group)

Opposite results were obtained after the administration of Ngb-carried lentivirus into the bilateral hippocampus (Fig. 4). Rats were randomly subjected to Sham operation or tGCI with or without hypoxia 14 days after the administration of Ngb-carried lentivirus (referred to as Lenti-*Ngb*) or scrambled lentivirus vector (referred to as Lenti-control) (Fig. 4A). As shown by immunofluorescence, Ngb was colocalized with GFP in Lenti-*Ngb*-administrated rats at 7 days after reperfusion (Fig. 4C). Similarly, no neurotoxic effects were observed on the pyramidal neurons in the CA1 of Sham-operated rats with lentivirus administration (Fig. 4D, a–h). Also, Ngb expression significantly increased in Sham-operated and tGCI rats after Lenti-*Ngb* administration (Fig. 4G). Meanwhile, Lenti-*Ngb* administration markedly ameliorated cell damage and neuronal loss, compared to Lenti-control administration after tGCI (Fig. 4D, i–p; Fig. 4E, F). However, no additive effect on neuroprotection was observed when either Lenti-*Ngb* or HPC was applied (Fig. 4D, q–x; Fig. 4E, F).

### The upregulation of Ngb induced by HPC increased the expression of membranous Atp1b1 in the hippocampal CA1 after tGCI

**Fig. 5:**
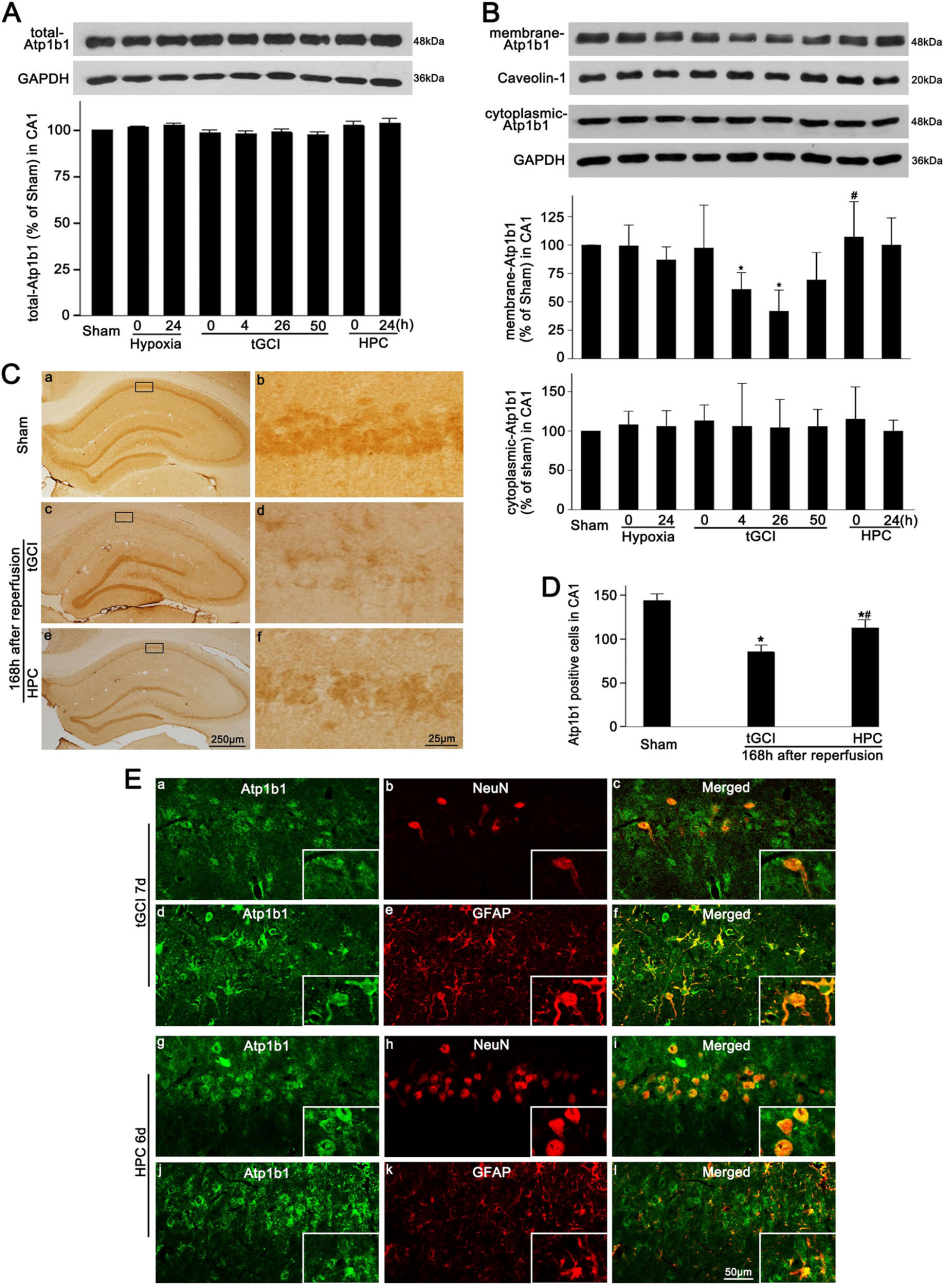
Effect of HPC on the protein expression and cellular localization of Atp1b1 in the CA1 after tGCI. **A** Representative immunoblots of Atp1b1 expression in total proteins in the CA1. The histogram presents the quantitative analyses of Atp1b1 levels (n = 3 in each group). **B** The immunoblot analysis of Atp1b1 protein levels in the cytoplasmic and the membrane fraction from CA1. Data are expressed as percentage of value of Sham-operated animals. Each bar represents the mean ± S.D. *p < 0.05 vs. Sham-operated animals, ^#^*p* < 0.05 vs. tGCI group (n ≥ 3 in each group). **C** Immunohistochemistry of Atp1b1 in the rat brains. Representative images show the Sham-operated group (a and b), 168 h after reperfusion of the tGCI groups (c and d), and 168 h after reperfusion of the HPC groups (e and f), respectively. Boxes indicate the magnified regions displayed in the right panel. **D** Quantitative analysis of Atp1b1-positive cells in the CA1. Each bar represents the mean ± S.D. *p < 0.05 vs. Sham-operated animals and ^#^*p* < 0.05 vs. tGCI group (n = 6). **E** Representative photomicrographs of fluorescent double staining show that Atp1b1 (green) was mainly colocalized with GFAP (red) (d-f) rather than NeuN (red) (a-c) in the CA1 at 168 h after tGCI. However, Atp1b1 colocalized with NeuN (g-i), but not GFAP (red) (j-l) at 168 h after tGCI with hypoxia. Scale bar: 50 μm

As shown in Fig. 5A, compared with the Sham-operated group, the tGCI groups with or without hypoxia showed no significant differences in the level of total Atp1b1. We thereafter further measured the membranous Atp1b1 expression. Notably, membranous Atp1b1 exhibited a similar trend compared to Ngb level, whereas there were no significant differences in the level of cytoplasmic Atp1b1 among the Sham-operated, hypoxia, tGCI, and HPC groups (Fig. 5B). Although the level of total Atp1b1 had no remarkable change at 0–50 h of reperfusion of tGCI with or without hypoxia, the number of Atp1b1-immunopositive cells significantly reduced at 168 h after tGCI. However, HPC induced an increase in the number of Atp1b1-positive cells (Fig. 5D). These Atp1b1-positive cells with astrocytes-like appearance were seen to be scattered over the stratum radiatum and oriens layer (Fig. 5C). Double-label immunofluorescence studies also confirmed that these cells were mainly colocalized with GFAP (Fig. 5E).

**Fig. 6:**
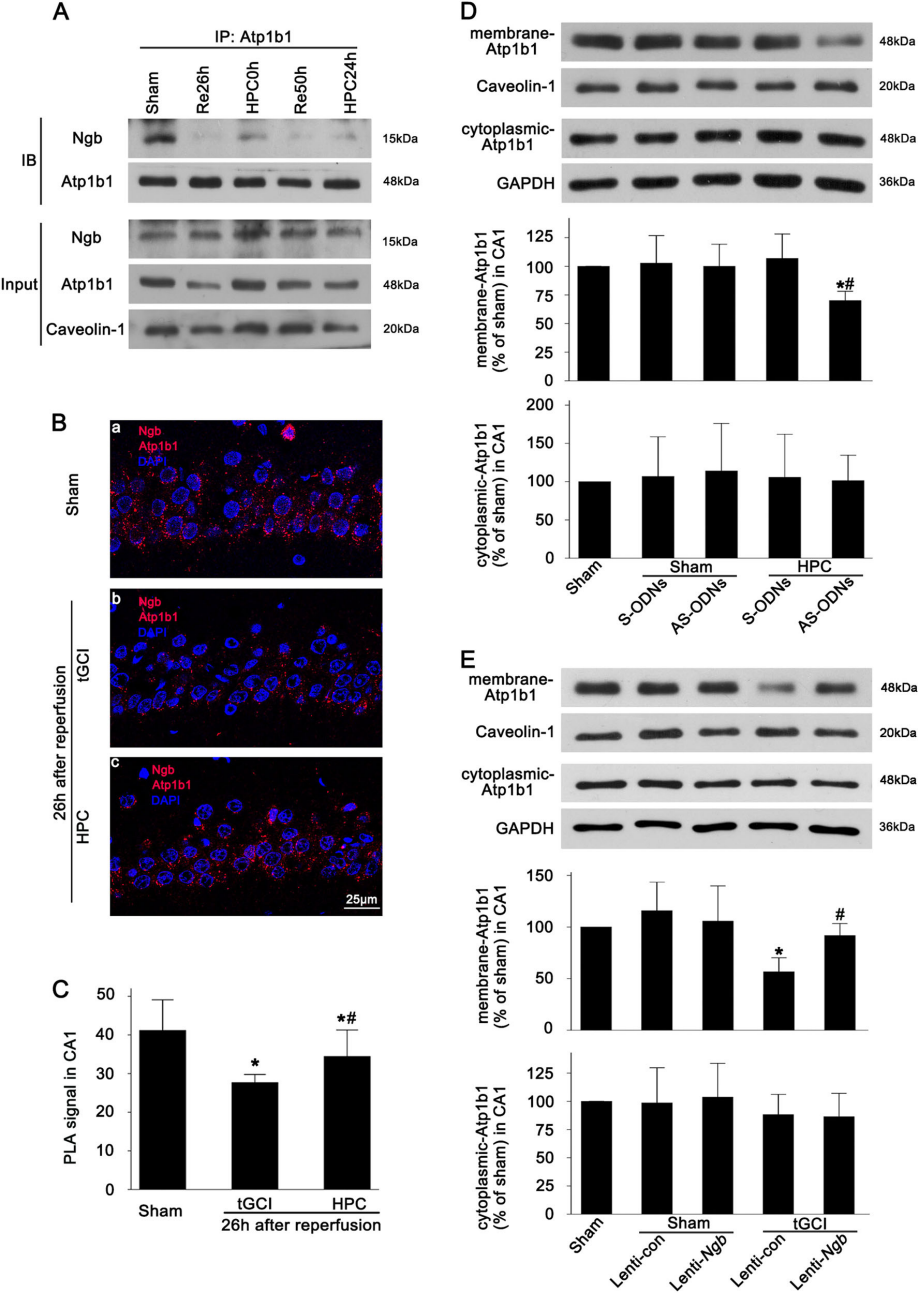
The regulation of Ngb to membrane protein of Atp1b1 in the CA1 after tGCI with or without HPC. **A** Immunoprecipitation blots showing the Ngb–Atp1b1 complex in the CA1 of the tGCI and the HPC groups. Atp1b1 was immunoprecipitated (IP) using anti-Atp1b1 antibody. Ngb was detected by western blot (WB). The experiments were repeated twice (*n* = 3 in each group). **B** Representative photomicrographs of proximity ligation assay showed the interaction between Ngb and Atp1b1 in the Sham-operated group (a), 26 h after reperfusion of the tGCI groups (b), and 26 h after reperfusion of the HPC groups (c). Dots (red) indicate Ngb–Atp1b1 complexes. Scale bar: 25 μm. **C** Quantitative analysis of PLA signal in CA1. Each bar represents the mean ± S.D. *p < 0.05 vs. Sham-operated animals and ^#^*p* < 0.05 vs. tGCI group at the same time point. **D** Immunoblot analysis of Atp1b1 in the cytoplasmic and the membrane fraction from CA1 of Sham and HPC groups with injection of Ngb ODNs. The values are expressed as percentage of value of Sham-operated animals. *p < 0.05 vs. Sham-operated animals and ^#^*p* < 0.05 vs. HPC group with S-ODNs administration (*n* ≥ 3 in each group). **E** Immunoblot analysis of Atp1b1 in the cytoplasmic and the membrane fraction from Sham-operated and tGCI group with injection of Lenti-control or Lenti-*Ngb*. Data are expressed as percentage of the value of Sham-operated animals. *p < 0.05 vs. Sham-operated animals, ^#^*p* < 0.05 vs. Sham-operated group with injection of Lenti-control, and ^&^*p* < 0.05 vs. HPC group with injection of Lenti-control (n ≥ 3 in each group)

We further examined the interaction between Ngb and membranous Atp1b1 in rats after tGCI with or without HPC by co-immunoprecipitation assay. The results revealed a detectable interaction between Ngb and membranous Atp1b1 after tGCI, though the strength of this interaction was weaker than that in the Sham-operated group. Moreover, HPC enhanced the strength of Ngb–Atp1b1 interaction, compared to the tGCI group (Fig. 6A). These observations were further confirmed by the proximity ligation assay (PLA) (Fig. 6B, C). Intriguingly, PLA signal was not only observed at the membrane, but also in the cytoplasm of the cells with typical neuronal appearance in the pyramidal cell layer of CA1. Afterwards, the regulatory effects of Ngb on the membranous and the cytoplasmic Atp1b1 were tested when Ngb expression was downregulated or upregulated. The results showed that the downregulation of Ngb with AS-ODNs in HPC rats led to a decrease in membranous Atp1b1, contrary to an increase in membranous Atp1b1 when Ngb was overexpressed with Lenti-*Ngb* in tGCI rats, whereas the regulation of Ngb expression had no impact on the level of cytoplasmic Atp1b1 (Fig. 6D, E).

### The upregulation of Ngb induced by HPC alleviated the levels of ROS and the glutathionylation of Atp1b1 in CA1 after tGCI

**Fig. 7:**
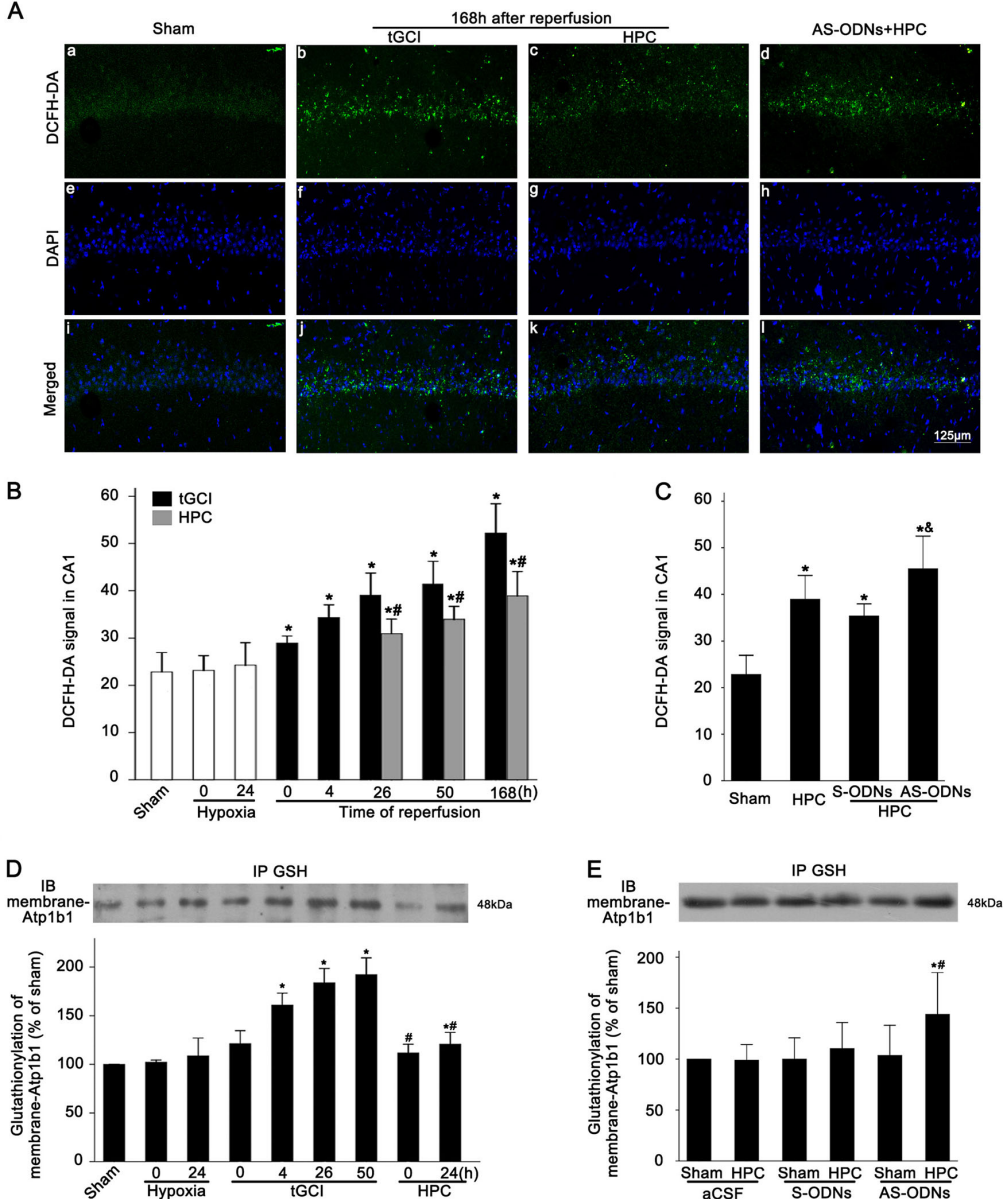
Effect of HPC on the level of ROS and the glutathionylation of membrane Atp1b1 after tGCI in CA1. **A** ROS level was evaluated with DCFH-DA in Sham, tGCI, and HPC groups with or without administration of Ngb ODNs. Representative photomicrographs show that ROS fluorescence (green) is distributed around DAPI (blue). **B**, **C** Quantitative analysis of DCFH-DA signal in the CA1 subregion. Each bar represents the mean ± S.D. *p < 0.05 vs. Sham-operated animals, ^#^*p* < 0.05 vs. tGCI group, and ^&^*p* < 0.05 vs. HPC group with injection of S-ODNs (*n* = 6 in each group). Scale bar: 125 μm. **D** Immunoprecipitation blots showing the glutathionylated Atp1b1 in CA1 of ischemic and hypoxic postconditioned rats. **E** Immunoprecipitation blots showing the glutathionylated Atp1b1 in the CA1 of Sham and HPC group with or without administration of Ngb ODNs. Glutathionylated proteins was immunoprecipitated (IP) using anti-GSH antibody. Membranous Atp1b1 was detected by Immunoblot (IB). The experiments were repeated twice (*n* = 3 in each group)

To elucidate whether the free radical scavenging capacity of Ngb is involved in the neuroprotective mechanisms of HPC against I/R injury, we examined the ROS level in CA1 with 2′,7′-dichlorofluorescein diacetate (DCFH-DA). The results showed a low level of ROS production at baseline in the Sham-operated group (Fig. 7A, a, e, i). However, ischemic stimuli were clearly observed to have triggered a robust generation of ROS. As shown in Fig. 7B, DCFH-DA signal in CA1 increased progressively and peaked at 7 days of reperfusion after tGCI. On the contrary, HPC efficiently decreased the ROS level, compared to the tGCI groups (Fig. 7A, B). Even so, the effect of HPC on the reduction of ROS was attenuated by the knockdown of Ngb with Ngb AS-ODNs (Fig. 7A, d, h, l, and Fig. 7B).

Given that the alteration of intracellular ROS affected the NKA activity by regulating the glutathionylation of Atp1b1, we measured the glutathionylation of Atp1b1 in the CA1 of tGCI and HPC groups with or without Ngb downregulation, respectively. As expected, the glutathionylation of Atp1b1 was upregulated after tGCI and downregulated by HPC (Fig. 7C), concomitant with the dynamics of ROS in the CA1 subregion. Moreover, the suppression of HPC on the glutathionylation of membranous Atp1b1 was impeded after downregulating Ngb expression with Ngb AS-ODNs (Fig. 7D).

### The upregulation of Ngb mediates the neuroprotection of HPC against tGCI via preserving the activity of Na^+^/K^+^ ATPases

**Fig. 8:**
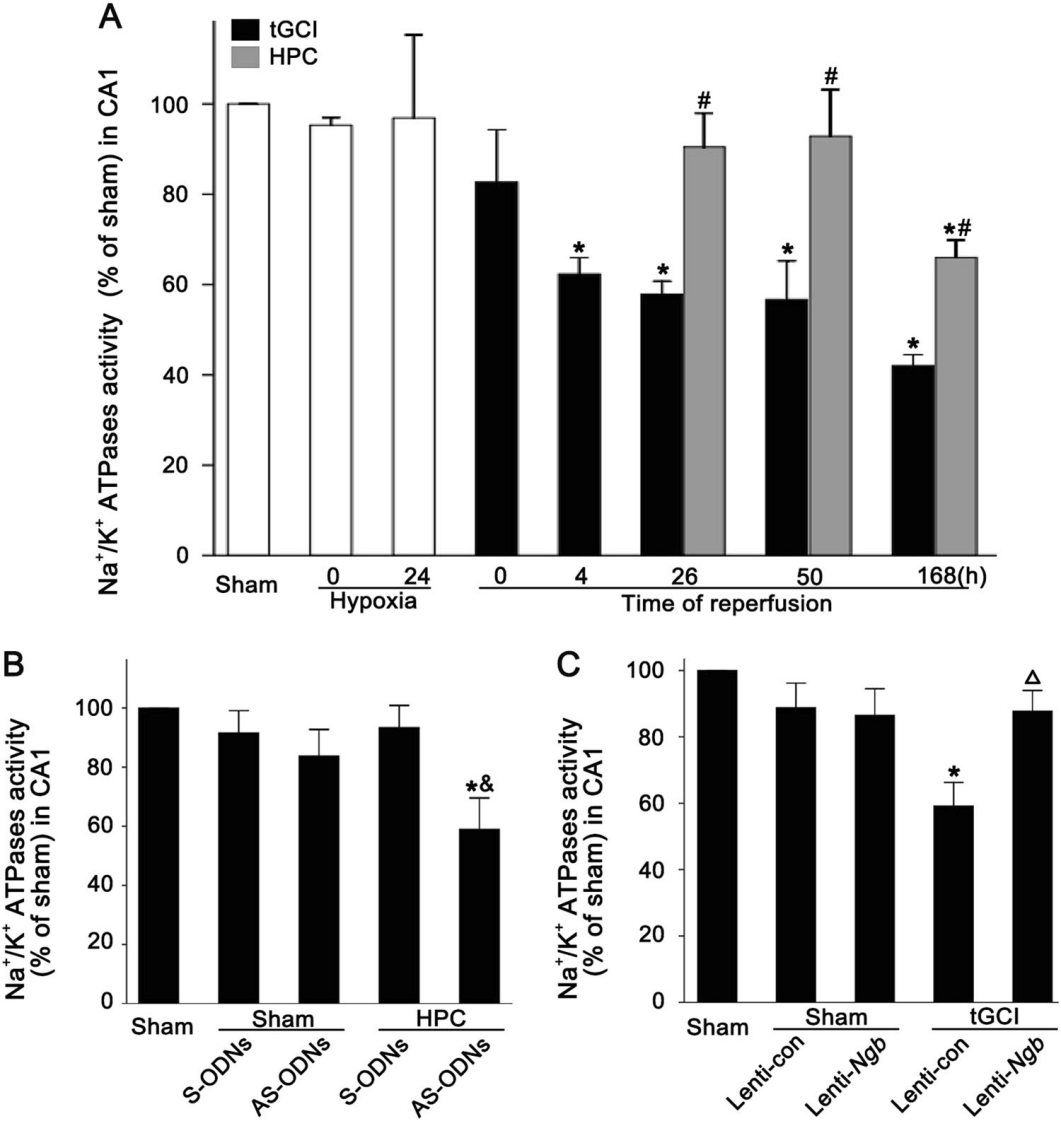
Effect of HPC on the activity of Na^+^/K^+^-ATPase in CA1 after tGCI. **A** Quantitative analysis of the NKA activity in the CA1 subregion in Sham, tGCI, and HPC groups. **B** Quantitative analysis of the NKA activity in the Sham and the HPC groups with or without ODNs administration. **C** Quantitative analysis of the NKA activity in the Sham and the tGCI groups with or without lentiviral administration. Data are expressed as percentage of the value of Sham-operated animals. Each bar represents the mean ± S.D. *p < 0.05 vs. Sham-operated animals, ^#^*p* < 0.05 vs. tGCI group, ^&^*p* < 0.05 vs. HPC group with injection of S-ODNs, and ^△^*p* < 0.05 vs. tGCI group with injection of Lenti-control (*n* ≥ 4 in each group)

Next, we examined the activity of NKA after tGCI with or without HPC and the effect of Ngb on the activity of NKA. No differences were observed in the NKA activity in Sham-operated and Sham-operated hypoxia-treated groups. Nevertheless, compared to Sham-operated rats, the NKA activity in the tGCI groups significantly reduced at 4 h after reperfusion and maintained a gradual decrease until 7 days (Fig. 8A). In contrast, HPC significantly preserved the NKA activity compared with tGCI rats, which was abrogated when Ngb was downregulated via Ngb AS-ODNs administration (Fig. 8B). Meanwhile, the Ngb overexpression with Lenti-*Ngb* reversed the inhibition of NKA activity in tGCI rats (Fig. 8C).

## Discussion

This study demonstrates that HPC activated endogenous neuroprotective mechanisms through upregulating Ngb expression in the CA1 subregion after tGCI. Ngb has been proven to be essential, highly conserved, and beneficial to neurons^38^. However, some previous studies reported that Ngb exerts no neuroprotective effect in mild traumatic brain injury^39^ and Ngb deficiency reduces cerebral infarct volume after permanent cerebral artery occlusion^20^. In this study, HPC induced upregulation of the Ngb expression in the CA1 and mediated neuroprotective effects after tGCI. To further confirm the involvement of Ngb in HPC-mediated neuroprotection, we silenced Ngb expression in CA1 with Ngb AS-ODNs and overexpressed Ngb with Ngb-carried lentivirus. As expected, Ngb overexpression effectively ameliorated post-ischemic neuronal death in CA1, whereas the silencing of Ngb expression abolished the neuroprotection of HPC. This neuroprotective role of Ngb after cerebral ischemia may depend on several distinct factors. First, there are significantly different expression levels of Ngb in different conditions. Physiologically, the concentration of Ngb in the brain is quite low (<0.01% of the total protein)^10^. Therefore, neuroprotection of the endogenous expressed Ngb is negligible^40^. However, under cerebral ischemia, the expression of Ngb in CA1 is reduced, while the upregulation or overexpression of Ngb is reported to promote neuronal survival^17,41^. Consistent with the previous studies, our results revealed that Ngb upregulation induced by HPC ameliorated neuronal injury in the CA1 after tGCI. Second, the distinct cell types may have an impact on the expression of Ngb. Our study demonstrated that Ngb level decreased at 26 h in the neurons after tGCI. However, activated astrocytes induced the upregulation of Ngb at 168 h after tGCI, which may have contributed to delayed cell death after ischemia. In contrast, HPC enhanced the expression of Ngb in the neurons, which consequently promoted neuronal survival after ischemia. Last, the anatomical and subcellular distribution of Ngb was clearly revealed to be associated with its neuroprotection. In a study from Shang et al., Ngb expression increased in the cerebral cortex and decreased in the hippocampus after transient global forebrain ischemia in the gerbil brain^42^. Differently, neither the tGCI nor the HPC group exhibited a significant change in the number of Ngb-positive cells in the cortex. These results suggested that the hippocampus is more vulnerable than the cortex to tGCI. High expression of Ngb could protect the neurons from ischemic damage. Furthermore, Ngb was recognized only in the cytoplasm previously. However, with immune localization, cell fractionation, and electron microscopy, Ngb has been found in the cytosol, inner wall of the mitochondria, and nuclei of the neurons^12,43^. Noteworthily, Ngb can shift between cellular organelles to change its compartmentation and concentration upon physiopathological situation^12,44^.

The upregulation of Ngb gene expression under hypoxia was reported to be regulated by several transcription factors such as nuclear factor κB (NF-κB), nuclear factor erythroid 2-related factor 2 (Nrf-2), and hypoxia-inducible factor-1α (HIF-1α)^45,46^. Intriguingly, our previous research showed a significant increase of HIF-1α and its downstream target vascular endothelial growth (VEGF) in the HPC groups, compared with the tGCI rats^6^. Considering this point, we speculated that the upregulation of Ngb protein by HPC might be associated with the regulation of HIF-1α and VEGF. Acting as Ngb inducers, both HIF-1α and VEGF can enhance the expression of Ngb^47,48^. Haines et al. further confirmed this connection between HIF-1α and Ngb induction. Under the condition of knockdown or overexpression of HIF-1α, the expression of Ngb decreased or increased in cultured neural cells^47^. In addition, the overexpressed Ngb can inhibit the apoptotic cascade by promoting the activation of the pro-survival Akt pathway and regulating the induction of the anti-apoptotic Bcl-2 protein network^11,49^. Previously, we had reported that the activation of Akt/FoxO pathway by HPC contributed to the induction of neuroprotection against tGCI^3^, suggesting that the upregulation of Ngb mediated by HPC may be related to the activation of Akt pathway.

Several possible mechanisms of neuroprotection conferred by Ngb have been proposed, including oxygen sensing, ROS scavenging, regulation of signal transduction, and maintenance of the mitochondrial function^37,50–52^. Mitochondrial impairment resulted from cerebral ischemia, and reperfusion was reported to cause rapid increase of ROS^53^, and the capability of Ngb in relieving oxidative stress has been well documented^11,38^. On one hand, Ngb can be bound to the mitochondrial complex III subunit Cyc1, a component of mitochondrial respiratory chain and a major source of ROS production. The interaction between Ngb and Cyc1 can lead to the inhibition of the mitochondrial complex III activity and the suppression of subsequent ROS generation^18,40^. On the other hand, Ngb could bind to O_2_ with higher affinity and change the oxidation state of its heme iron from ferric to oxygenated ferrous form to efficiently scavenge a variety of ROS^51^. Our study showed the gradually increased level of ROS in the CA1 over time after the reperfusion of tGCI and reduced level in the HPC groups. Furthermore, downregulation of the Ngb expression with Ngb AS-ODNs reversed the effect of HPC on the suppression of the ROS level.

Oxidative stress can activate pro-apoptotic signaling pathway and initiate cell damage cascade^52,54,55^. Notably, oxidative stress played a role in the regulation of NKA that is essential for a number of vital cell functions^35,56,57^. Previous research showed that oxidation regulated the activity of NKA through inducing reversible glutathionylation of Atp1b1^36^. In the current study, we found that glutathionylation of Atp1b1 gradually enhanced after tGCI, but reduced in HPC rats, exhibiting the same trend as the ROS level. Additionally, the effect of HPC on the reduction of Atp1b1 glutathionylation was dampened when Ngb was downregulated. Consequently, the reduction of Atp1b1 glutathionylation after HPC might be caused by Ngb upregulation and subsequent ROS suppression. Noteworthily, the interaction between ROS and NKA causes a conformational change of NKA^58^. Meanwhile, the production of ROS inhibits the activity of NKA. In turn, the activation of NKA signaling cascade stimulates more generation of ROS with an amplification loop^59^. Previously, we have observed a reduction in the activity of NKA and the protein level of NKA α1 subunit after tGCI. Furthermore, hypoxic preconditioning enhanced the recovery of NKA activity by preserving NKA α1 subunit, thereby reduced DNA fragmentation after tGCI^60^. The current study reconfirmed the suppressed activity of NKA in the CA1 after ischemia, accompanied by an increase of ROS production. Also, HPC maintained the activity of NKA and reduced ROS through the upregulation of Ngb in CA1, thus exerting neuroprotection against tGCI.

Proteins that interact with Ngb, including those involved in ionic homeostasis maintenance, energy metabolism, mitochondrial function, and signaling pathways for cell survival and proliferation, may provide important clues for the exploration of the functions played by Ngb^40^. Interestingly, Atp1b1 is one of the various putative interacting partners of Ngb and their interaction had been verified in the cultured mouse cortical neuron^30^. Consistent with the expression pattern of Ngb, the level of membranous Atp1b1, rather than total or cytoplasmic Atp1b1, decreased in the CA1 after tGCI, but evidently increased with HPC. These results suggested that the increase of Atp1b1 in the membranous fraction did not result from the relocalization from cytoplasmic fraction. Additionally, the overexpression of Ngb with Lenti-*Ngb* increased membranous Atp1b1 in the CA1 after tGCI, whereas Ngb downregulation with Ngb AS-ODNs impeded the augment of membranous Atp1b1 and the rescue of the NKA activity resulting from HPC. Although the physiological role of Ngb–Atp1b1 interaction remains to be investigated, we speculate that the role of Ngb in maintaining membranous Atp1b1 may result from its direct interaction with Atp1b1. In our research, we did observe the interaction between Ngb and Atp1b1 in CA1, which was significantly enhanced by HPC after tGCI. It was reported that increased ROS can oxidize NKA α/β subunits, thereby promoting its susceptibility to degradation^35,56^. Hence, the maintenance of membranous Atp1b1 after HPC is more likely to be the inhibition of the degradation of Atp1b1, which may depend on HPC-induced protection of Atp1b1 from ROS. Notably, with the binding properties and (pseudo-)enzymatic properties, Ngb is able to releases O_2_ to bind ROS and reactive nitrogen radicals (RNS) during cerebral ischemia^11,61^. Therefore, in addition to suppressing the ROS level, Ngb in the Ngb–Atp1b1 complex may bind ROS and shield Atp1b1 to block the oxidation of β subunit derived from ROS, thereby preserving membranous Atp1b1.

In conclusion, we have demonstrated the central role of Ngb in HPC-mediated neuroprotection against tGCI for the first time. Ngb effectively scavenged the overproduced ROS after cerebral ischemia and in turn prevented the inhibition of NKA activity that resulted from oxidative stress-modulated Atp1b1 glutathionylation. In addition, the direct interaction of Ngb with Atp1b1 in vivo may help maintain membranous Atp1b1, which is responsible for preservation of the NKA activity. Since Ngb can act as an endogenous neuroprotectant, in-depth identification of its functions and potential mechanisms would open up new areas of a non-pharmacological therapeutic intervention against cerebral ischemia.

## Materials and methods

This study was performed with the approval of the Animal Care and Use Committee of Guangzhou Medical University. Adult male Wistar rats weighting 220 to 280 g (Southern Medical University, Guangdong, China) were treated in accordance with the Animal Research: Reporting In Vivo Experiments (ARRIVE) guidelines. Animals were housed in a temperature-controlled (22 °C ± 1 °C) and 12 h light/dark cycle environment, fed with ad libitum food and water. All efforts had been made to minimize both the number of animals used and the suffering of the animals.

In this study, 719 rats were used, 20 of which in the tGCI group and 8 in the HPC group died during the procedure of tGCI, 8 in the tGCI group and 9 in the HPC group died during reperfusion after tGCI. Also, 9 died after intracerebroventricular injection of ODNs; another 6 died after lentiviral administration; two died during the procedure of hypoxia; another 6 rats died after anesthesia. Thirteen rats that convulsed during ischemia and 7 during 72 h after tGCI were also excluded.

### Experimental models

Transient global cerebral ischemia model was produced by a four-vessel occlusion method^62^. Concisely, animals were anesthetized intraperitoneally with 10% chloral hydrate (350 mg/kg). Preoperation skin preparation was performed around the incision. Bilateral vertebral arteries were electrocauterized and blocked after exposing the alar foramen. Common carotid arteries were isolated and assembled with a teflon/silastic occluding device without blocking the carotid blood flow. Global ischemia was induced in awake animals 24 h after surgery by occluding both common carotid arteries for 10 min. Rats that found mydriasis and lost righting reflex within 1 min were enrolled for later experiments. Rectal temperature was maintained at ~37–38 °C throughout the whole procedure. Animals in the Sham group received the same surgical process, except occluding common carotid arteries. All operations were conducted under aseptic conditions.

Rats were exposed to systemic hypoxia 24 h after tGCI for the postconditioning process^63^. Briefly, rats were placed in a sealed plastic chamber of 9000 cm^3^ with a mixed gas composed of 8% O_2_ and 92% N_2_ for 120 min at the temperature of 23–25 °C.

### Immunohistochemistry

The animals were sacrificed at 0, 4, 26, 50, and 168 h after reperfusion with or without HPC (*n* = 6 in each group), respectively. Single-label immunohistochemistry was detected by the avidin–biotin–peroxidase complex (ABC) method^63^. Briefly, free-floating sections were soaked in 3% H_2_O_2_ for 30 min and then 5% normal serum for 1 h, followed by incubation overnight at 4 °C with rabbit anti-Ngb primary antibody (diluted 1:300; Proteintech Group, Inc. Chicago, IL) and a mouse antibody to Atp1b1 (diluted 1:100; Millipore, Bedford, MA). Afterwards, the sections were incubated with biotinylated secondary immunoglobulin G antibody for 2 h at room temperature. Subsequently, the sections were treated with ABC for 30 min at room temperature. Immunoreaction was visualized using 0.05% diaminobenzidine and 0.01% H_2_O_2_. Immunopositive cells, in which the reaction product was present within a clear and regular-shaped cytoplasmic or nuclear border, were quantified under a light microscope (×660). The total number of immunoreactive cells was counted by the total number of four non-repeated random fields (0.037 mm^2^ per field × 4 = 0.148 mm^2^ total) in the CA1 regions.

Double-fluorescent immunohistochemistry was conducted to examine the cell types and the subcellular location of the expression of Ngb and Atp1b1. MAP-2, NeuN, and GFAP were used to identify the neuronal cell bodies and the dendrites, nuclei of neurons and astrocytes, respectively. Double-fluorescent immunohistochemistry was performed as described previously^63^. Antibodies used in this part include the mouse and rabbit anti-NeuN (diluted 1:3000; Millipore, Bedford, MA, USA), mouse and rabbit anti-GFAP (diluted 1:4000; Millipore), Cy3-conjugated goat anti-mouse and anti-rabbit IgG antibody (diluted 1:100; Millipore, Bedford, MA), FITC-conjugated goat anti-rabbit antibody (diluted 1:100; Abcam, Inc., Cambridge, UK), and FITC-conjugated goat anti-mouse antibody (diluted 1:100; Millipore, Bedford, MA). Slides were analyzed with a confocal laser microscope (XP8, Leica Microsystems, Wetzlar, Hessen, Germany). The total number of Ngb-positive neurons or astrocytes was also counted within the four non-repeated random fields (0.037 mm^2^ per field × 4 = 0.148 mm^2^ total) in the CA1 regions.

### Western blotting

The animals were sacrificed at 0, 4, 26, and 50 h after reperfusion with or without HPC (*n* = 3 in each group), respectively. Rats were killed under chloral hydrate anesthesia. The brain tissue was incised into 2-mm coronal slices with a brain matrix, and the CA1 regions of bilateral hippocampi were quickly divided under the stereomicroscope. The protein extraction and western blotting procedure were performed as described previously^63^. In order to detect the level of Atp1b1, membranous and cytosol protein were extracted with a membrane and cytosol protein extraction kit (Beyotime Biotechnology). The primary antibodies included rabbit anti-Ngb (diluted 1:200; Proteintech Group), mouse anti-Atp1b1 (diluted 1:5000; Millipore), mouse anti-GAPDH (diluted 1:10,000; Proteintech Group), and rabbit anti-Caveolin-1 (diluted 1:2000; Abcam, Inc., Cambridge, UK). Densitometric analysis for the quantification of the bands was performed with the image analysis software (Quantity One, Bio-Rad Laboratories, Inc., Hercules, CA). Relative optical densities of protein bands were calibrated with GAPDH or Caveolin-1 and normalized to those in the Sham-operated rats.

### Assessment of cellular damage

Animals were sacrificed at 7 days after tGCI, followed by perfusing intracardially with 0.9% saline and 4% paraformaldehyde in phosphate-buffered saline (PBS). The brain tissues were postfixed in 10, 20, and 30% sucrose in the same fixative for cytoprotection. After postfixation, the brains were frozen at −20 °C and sliced into coronal 30-μm-thick sections with cryotome (Leica, Wetzlar, Hessen, Germany). Sections selected from the dorsal hippocampus (between AP 4.8 and 5.8 mm, interaural or AP −3.3 to 3.4 mm, bregma) were used for Nissl, NeuN, and FJ-B staining, which was carried out according to a protocol described in our previous study^3^.

Nissl- and NeuN-stained sections were observed under a light microscope with ×660 magnification. The survived cells displayed well-stained Nissl bodies, whereas the damaged cells were either swollen with loss of stainable Nissl material or necrotic with deeply staining dendrites fragmented. FJ-B-stained images were observed with a fluorescence microscope (Leica Microsystems, Wetzlar, Hessen, Germany). Cell counts were conducted by densities, as previously described^64^. Cells in the CA1 pyramidal layer were quantitatively analyzed within three non-repeated rectangular areas of 0.037 mm^2^. Data were quantified bilaterally in sections from each brain and assessed double-blindedly. Also, four sections for each animal were evaluated.

### Drug administration

Sequences for Ngb AS- and S-ODNs were referenced to the report by Sun et al.^41^. The sequence of Ngb AS-ODNs was: 5′-TCCGGGCGCTCCAT-3′ (nucleotides 90-77) and Ngb S-ODNs was: 5′-ATGGAGCGCCCGGA-3′ (nucleotides 77–90). AS-ODNs and S-ODNs were synthesized with a phosphorothioate backbone, purified with ULTRAPAGE (Sangon, Inc. Shanghai, China), and dissolved with aCSF. Solution in volume of 8 µl (16 µmol/l) was injected over a 10-min period each time intracerebroventricularly 30 min before tGCI and HPC. For Sham-operated rats, ODNs or aCSF was administrated twice at the same time point in 2 days after operation.

### Lentivirus construction and lentiviral administration

Plasmids containing the sequence of rat *Ngb* (GenBank accession number NM_85382), CGCCACCATGTGCGTTGACGCCGTGCCCACGCCTCGAGGGTCCCATCACTGCGTCCCGCGACTCTCCTGGGAGAGAGAGAGTATGGAGCGCCTAGAGTCAGAGCTGATCCGGCAGAGCTGGCGGGCAGTGAGCCGCAGCCCTCTGGAACATGGCACTGTCCTGTTCTCCAGGCTCTTCGCCCTGGAACCCAGCCTGCTGCCTCTCTTCCAGTACAATGGCCGCCAGTTCTCCAGTCCTGAGGACTGTCTCTCCTCTCCAGAATTCCTGGACCACATTAGGAAGGTTATGCTTGTGATTGATGCTGCTGTGACCAACGTAGAGGACCTGTCTTCACTGGAGGAGTACCTGGCCACCTTGGGCAGGAAGCATCGGGCAGTGGGAGTGAGGCTCAGCTCCTTCTCGGTGGGTAGTGGAACAGTAGGTGAGTCCCTGCTCTACATGCTGGAGAAGTGCCTGGGTCCCGACTTTACGCCGGCTACAAGGACCGCCTGGAGCCAACTCTACGGAGCTGTGGTGCAAGCCATGAGCCGAGGCTGGGACGGGGAG, and a negative control sequence were designed by Genechem (Shanghai, China). The sequence was inserted into *Age*I and *BamH*I sites of the Ubi-MCS-3FLAG-CMV-EGFP (GV365) lentiviral vector, according to the manufacturer’s instruction. The shuttle vector and viral packaging system were cotransfected into HEK293T cells to produce recombinant lentiviruses using Lipofectamine 2000 (Invitrogen, San Diego, CA, USA). Then HEK293T cells were used for viral infection. The infection efficiency was greater than 80%, as monitored with GFP protein expression. After 48 h of infection with Lenti-*Ngb*, the cells were harvested and the total RNA were extracted to examine the expression of Ngb mRNA. The titers were approximately 1 × 10^9^ TU/ml.

A total of 5 μl volume (1.25 μl virus diluted by 3.75 μl enhanced solution) containing 1.25 × 10^9^ TU/ml of particles was injected into the bilateral hippocampal CA1 region (3.5 mm posterior to bregma, 2.3 mm lateral to bregma, and 2.6 mm below the dura) using the RWD syringe and WPI infusion pump at a speed of 30 μl/h. Rats were accepted for four-vessel occlusion surgery or Sham-operation 14 days after administration.

### Membrane protein extraction

The animals were sacrificed at 0, 4, 26, and 50 h after reperfusion with or without HPC, respectively. Tissue samples of the hippocampal CA1 subregion were isolated as described previously^8^. Membrane protein of the CA1 subregion was extracted via the tissue membrane protein prep kit (GenMed Scientifics Inc. USA), according to the manufacturer’s protocol. After washed by Reagent A, 1 g tissues were homogenized with 4 ml working solution containing 4 ml of Reagent B and 5 μl Reagent C, followed by centrifugation at 1000 g for 10 min at 4 °C. Then 1 ml of Reagent B was added in the precipitates, followed by centrifugation again. Supernatants after twice centrifugation were incubated with 5 ml of Reagent E on ice for 60 min, followed by centrifugation at 20,000 × *g* for 60 min at 4 °C. After vibrated for 15 s with 1 ml of Reagent F, the precipitates were stored at –80 °C before the use in co-immunoprecipitation assay.

### Co-immunoprecipitation

For co-immunoprecipitation assay, 150 μg membrane proteins were incubated with mouse anti-Atp1b1 monoclonal antibody (diluted 1:200; Millipore, Bedford, MA) at 4 °C overnight. The protein/antibody complex was added to packed protein G agarose beads (Millipore, Bedford, MA, USA). Following 4 h of incubation at 4 °C, the complex was washed five times with PBS containing 1% Tween. Then immunocomplexes were separated by incubation with protein G agarose beads and were resolved by SDS-PAGE. Immunoblot assay was performed with rabbit anti-Ngb antibody (diluted 1:200; Proteintech Group) and mouse anti-Atp1b1antibody (diluted 1:5000; Millipore, Bedford, MA).

### Proximity ligation assay

Proximity ligation assay was carried out with Duolink® In Situ Red Starter Kit Mouse/Rabbit (DUO92101, Sigma-Aldrich), according to the manufacturer’s protocol. Briefly, the sections selected from the dorsal hippocampus were soaked in blocking solution for 1 h and then incubated overnight at 4 °C with rabbit anti-Ngb antibody (diluted 1:100; Proteintech Group, Inc. Chicago, IL) and mouse anti-Atp1b1 (diluted 1:20; Millipore, Bedford, MA). After labeling with PLUS and MINUS PLA probes for 1 h, the sections were incubated with Ligation-Ligase solution and Amplification-Polymerase solution at 37 °C. PLA signals were detected with a fluorescence microscope (Leica Microsystems, Wetzlar, Hessen, Germany) and the images were analyzed with ImageJ.

### ROS measurement

A slight modification of ROS measurement described by Matsushima et al. was used^65^. The animals were sacrificed at 0, 4, 26, 50, and 168 h after reperfusion with or without HPC and 168 h after HPC with Ngb AS- and S-ODNs administration (*n* = 6 in each group), respectively. After harvest, the brain tissues were immediately stored at −80 °C for 2 h. Then the unfixed frozen brains were cut into 30-µm-thick sections and placed on glass slides. The oxidation-sensitive fluorescent dye DCFH-DA (4.44 μg/ml) (Molecular Probes, Inc. Oregon, USA) was applied to each tissue section, and then the sections were coverslipped. The slides were incubated in a light-protected humidified chamber at 37 °C for 35 min. Green ROS fluorescence was observed with a fluorescence microscope (Leica Microsystems, Wetzlar, Hessen, Germany).

### Glutathionylation of Na^+^/K^+^ ATPase β_1_ subunit

The glutathionylation of Atp1b1 was detected according to the method in the previous research^34^. Briefly, the membrane proteins were mixed with anti-GSH antibody (diluted 1:100; Virogen, Watertown, MA) at 4 °C overnight to isolate the glutathionylated proteins. Then, the proteins were mixed with packed protein G agarose beads (Millipore, Bedford, MA, USA) which had been pre-washed by cold PBS (12,000 × *g*, 3 min, 3 times, 4 °C). After incubation at 4 °C for 4 h, the beads were washed with cold PBS (12,000 × *g*, 5 min, 5 times, 4 °C). Then 15 μl of loading buffer was added to the beads and the mixture was heated at 37 °C in a water bath for 15 min to be used for immunoblotting. Last, Atp1b1 in the immunoprecipitate was identified with western blotting to show the glutathionylation of Atp1b1.

### Measurement of the Na^+^/K^+^-ATPase activity

With minor modifications, the Na^+^/K^+^-ATPase activity was measured according to the manufacturer’s instructions (Nanjing Jiancheng Bioengineering Institute, Nanjing, China), as described previously^60^. Briefly, the tissues from the CA1 subregion of rats sacrificed at 0, 4, 26, 50, and 168 h after reperfusion with or without HPC, Sham-operated or 26 h after reperfusion in the HPC group with Ngb AS- or S-ODNs administration, and Sham-operated or 26 h after reperfusion in the tGCI group with Lenti-control or Lenti-*Ngb* administration were homogenized in 0.86% sodium chloride solution. After centrifugation at 1000 rpm for 5 min, the supernatant was used for the reaction of Na^+^/K^+^-ATPase assay. All samples were run in duplicate. Specific activity of the enzyme was expressed as nmol Pi released per min per mg of protein. For the final calculation, the activity of Na^+^/K^+^-ATPase in each group was expressed as a percentage of the Sham-operated control.

### Statistical analysis

Data analysis was performed with Statistical Package for Social Sciences for Windows, version 13.0 (SPSS, Inc, Chicago, Illinois, USA). All variables were expressed as mean ± standard deviation. The statistical significance was determined by one-way analysis of variance (ANOVA) or two-tailed Student’s *t*-test. The differences were considered statistically significant when *p* < 0.05.
